# Chronic Obstructive Pulmonary Disease and Its Effect on Red Blood Cell Indices

**DOI:** 10.7759/cureus.36100

**Published:** 2023-03-13

**Authors:** Sara Tariq, Dina Ismail, Milan Thapa, Lakshmi Goriparthi, Roshini Pradeep, Khizer Khalid, Ayden Charlene Cooper, Gutteridge Jean-Charles

**Affiliations:** 1 Internal Medicine, Mayo Hospital, Lahore, PAK; 2 Internal Medicine, JC (Jean-Charles) Medical Center, Orlando, USA; 3 Family Medicine, University Hassan II of Casablanca Faculty of Medicine and Pharmacy, Casablanca, MAR; 4 Internal medicine, Monmouth Medical Center, Long Branch, USA; 5 General Surgery, Osmania Medical College, Hyderabad, IND; 6 Internal Medicine, Midwestern University Arizona College of Osteopathic Medicine, Phoenix, USA; 7 Internal Medicine, Advent Health & Orlando Health Hospital, Orlando, USA

**Keywords:** rbc structural alterations, secondary polycythemia, anemia of chronic disease (acd), rbc indices, copd: chronic obstructive pulmonary disease

## Abstract

Chronic obstructive pulmonary disease (COPD) constitutes a set of heterogeneous symptoms affecting millions of people worldwide. The associated comorbidities developing in COPD involve dysregulation in physiological pathways resulting from systemic inflammation in respiratory airways. In addition to mentioning the pathophysiology, stages, and consequences of COPD, this paper also defines red blood cell (RBC) indices such as hemoglobin, hematocrit, mean corpuscular volume, mean corpuscular hemoglobin concentration, red blood cell distribution width, and RBC count. It explains the role of RBC indices and RBC structural abnormalities with disease severity and exacerbations in COPD patients. Although many factors have been studied as a marker of morbidity and mortality for COPD patients, RBC indices have emerged as revolutionary evidence. Therefore, the effectiveness of evaluating RBC indices in COPD patients and their importance as a negative predictor of survival, mortality, and clinical outcomes have been debated through rigorous literature reviews. Furthermore, the prevalence, mechanisms of development, and prognosis of underlying anemia and polycythemia in COPD have also been evaluated, with anemia most significantly associated with COPD. Therefore, more studies should be conducted to address underlying anemia in COPD patients to lessen the severity and disease burden. Correcting the RBC indices in COPD patients remarkably impacts the quality of life and reduces in-patient admissions, healthcare resource utilization, and costs. Hence, it is noteworthy to understand the significance of considering RBC indices while dealing with COPD patients.

## Introduction and background

Chronic obstructive pulmonary disease (COPD) is one of the most disabling chronic diseases, with an increasing prevalence and death rates worldwide. Among other causes of mortality, COPD is the fourth leading cause of death in the United States [1]. Furthermore, COPD is associated with several comorbidities and complications as part of a systemic effect contributing to the severity of the illness. Many significant events can occur in the disease's natural history, potentially causing major comorbidities, economic burdens, and mortality [2]. These coexisting conditions are a direct effect of COPD evolution, involving chronic inflammation and oxidative stress as strong components in its pathogenesis [3]. The increase in reactive oxygen species (ROS) and inflammatory markers is a hallmark causing airway and lung damage in COPD patients [4]. However, they can have implications beyond the lung and reflect in almost all the systems, including musculoskeletal, metabolic, renal, cardiovascular, and psychiatric [5]. The hematological system is far from being spared with implications in hemorheology, coagulability, platelets, white blood cells (WBCs), red blood cells (RBCs), hemoglobin (Hb), and RBC indices [6].

It has been observed that overall derangement in RBC indices is associated with poor pulmonary function and disease severity in COPD [7]. Specifically, elevated red blood cell distribution width (RDW), lower mean corpuscular hemoglobin concentration (MCHC), and Hb levels are associated with increased disease severity and lower survival rates in patients with COPD [8-10]. In addition, RBC structural alterations have also been linked with advanced stages of COPD [11]. Therefore, RBC indices are emerging as robust predictor tools of COPD disease severity and progression. 

After establishing a diagnosis in COPD patients, predicting the prognosis, such as exacerbation or mortality, is critical. Therefore, multiple prognostic indicators have been tested. Although various studies focus on the role of WBC, C-reactive protein (CRP), and other inflammatory markers as prognostic factors of COPD, very few have highlighted RBC indices. Our review will evaluate the association of RBC indices such as Hb, hematocrit (HCT), MCHC, and RDW with COPD and assess their application as markers of COPD disease severity, exacerbation, mortality, and hospital readmission rates. 

## Review

COPD 

Prevalence

According to the Centers for Disease Control and Prevention (CDC) survey, COPD age-adjusted prevalence has remained unchanged from 2011 to 2020, but it is reported to be higher in women than men due to delayed diagnosis, increased susceptibility to tobacco smoke, and varied responses to treatment [12]. Chronic lower respiratory disease, primarily COPD, was the fourth most significant cause of death in the United States in 2018, with women’s death rates higher than men’s. COPD has been diagnosed in nearly 15.7 million Americans (6.4%). However, more than half of the people with impaired pulmonary function were unaware that they had COPD, suggesting that the actual figure is far more significant [12]. 

In 2019, the disease was projected to have killed 3.23 million people, according to the World Health Organization (WHO). The latter is known to kill more than 90% of people in low- and middle-income nations [13]. In addition, 12.5 million people were diagnosed with COPD in 2020, with trends higher in non-Hispanic Whites (6.2%), women (5.2%), and >65-year age groups (10.8%) compared to Blacks (4.7%), men (4.3%), and 45-64 age groups (6%) [14].

Pathophysiology

Chronic inflammation causing increased frequency of certain inflammatory cell types in distinct lung areas and structural alterations arising from repetitive injury and repair are pathological abnormalities associated with COPD [15]. Small airway disease and parenchymal destruction are caused by cigarette smoking or exposure to noxious chemicals, which cause inflammation in the lungs and airways of the bronchial tree [16]. Lung inflammation is likely to be further modified by oxidative stress and an abundance of proteinases [15].

Stages

COPD is classified into four severity levels by the Global Initiative for Chronic Obstructive Lung Disease (GOLD) staging system (based on post-bronchodilator forced expiratory volume in one second (FEV1)): stage I or mild has an FEV1 of ≥80%; stage II or moderate has an FEV1 of ≥50% and <80%; stage III or severe has an FEV1 of ≥30% and <50%, and stage IV has an FEV1 of <30% [16]. As the condition progresses, daily activities become more restricted, resulting in a lower quality of life and increased symptoms and exacerbations [17].

In 2011, revised GOLD guidelines included the ABCD assessment tool, categorizing COPD patients into four groups based on symptomatology, GOLD grades, and exacerbation history. This tool assesses the symptomatology by the COPD assessment test (CAT) and Modified Medical Research Council (mMRC) Dyspnea Scale, and exacerbation risk through GOLD grades (severity of airflow limitation) and history of exacerbation episodes. In 2017, GOLD updated the ABCD assessment tool by evaluating disease severity through symptom burden and exacerbation risk calculations independent of spirometric results [18].

*Consequences* 

COPD produces polycythemia secondary to erythrocytosis from hypoxia in advanced cases. However, several investigations have found that many COPD patients have anemia rather than erythrocytosis [19].

Anemia is an important complication that occurs during the clinical course of chronic diseases. It is thought to be caused by chronic inflammation and iron deficiency. Patients with COPD have a significant rate of iron deficiency [20]. The response to erythropoietin (EPO) in COPD also appears to be inhibited, especially as the disease progresses. Therefore, it could contribute to developing anemia in COPD patients [19]. Depending on the populations studied and the diagnostic techniques used to detect Hb levels, the prevalence of concomitant anemia in COPD patients ranges from 7.5% to 34%. The actual prevalence of anemia in COPD patients is unknown [21]. The chronic inflammatory processes in COPD promote deaths and membrane deformability of RBCs and alter erythropoiesis which is related to an increase in RDW [22].

Prognosis 

Several elements have been identified as COPD prognostic markers. FEV1, a measure of the severity of airflow limitation, is most often used. Once COPD has been diagnosed, predicting the prognosis, such as exacerbation or mortality, appears to be critical; yet, in some primary healthcare settings with an inferior approach to inspection, determining the prognosis seems to be a near-impossible task [23].

It is well recognized that COPD is associated with oxidative stress, chronic inflammation, and impaired iron metabolism. As a result, MCHC, RDW, and erythrocyte sedimentation rate (ESR) levels are thought to reflect the severity of COPD inflammation [22].

RDW has been identified as a potential predictor of all-cause death [10]. Mortality rates increased five-fold from the lowest to the highest quintile of RDW in the Third National Health and Nutrition Examination Survey of 15,852 adults [22].

Anemia and increased amounts of acute-phase proteins, fibrinogen, and immunoglobulin in the blood cause ESR to rise. COPD is frequently associated with hyperfibrinogenemia and anemia, especially in severe cases. As a result, if we consider COPD to be a systemic rather than just a respiratory disorder, ESR appears to be a promising choice for use as a prospective COPD severity index. In a study by Kanwal et al. in 2021, when an association between COPD and various RBC indices was observed, raised ESR was most significantly associated with COPD patients (p=0.001). It indicates the significance of monitoring ESR for understanding the progression and severity of the disease [22].

Pulmonary embolism is one of the most common and serious complications, which develops in hospitalized COPD patients with acute exacerbation episodes. A systematic review indicates its prevalence of 24.7% (p=0.001) in hospitalized COPD patients compared to patients admitted to the emergency department (3.3%) [24]. In a prospective study by Zorlu et al. in 2012, high ESR was independently linked to higher mortality from acute pulmonary embolism in 136 patients with acute pulmonary thromboembolism (hazard ratio 15.5) [22,25]. 

A study by Chambellan et al. in 2005 conducted on 2524 patients found that mortality decreased by 14% for every 5% increase in HCT [26].

RBC indices

Wintrobe was the first to introduce the red cell indices in 1929. Their role resides in determining erythrocyte size and Hb content. Traditionally, these indices help determine the etiology of anemia and are included in every full blood count (FBC). They are calculated by using the Hb level, HCT, and red blood cell (RBC) count through standard formulas. Nowadays, machines with automated cell counters can directly give us the values of red cell indices [27]. 

RBC Count

RBCs carry Hb, which plays a vital role in oxygen delivery to the tissues. A normal RBC count would be 4.7-6.1 million cells per microliter (cells/mcL) in men and 4.2-5.4 million cells/mcL in women [28].

Hemoglobin

Hb is a metalloprotein that contains iron and transports oxygen. The normal level of Hb for males ranges from 14 to 18 g/dL, and for females, Hb ranges from 12 to 16 g/dL. An Hb level below the normal range is called anemia [29].

Hematocrit

HCT, also known as packed cell volume, is a percentage of RBCs in the total blood volume, constituting RBCs and plasma. HCT in males ranges from 40% to 54%, whereas in females, HCT ranges from 36% to 48% [29].

Hb and HCT are determined by plasma volume based on whole blood. For example, in patients with severe dehydration, both Hb and HCT are higher than in euvolemic patients, contrary to patients with fluid overload, where Hb and HCT levels are lower [29].

Mean Corpuscular Volume (MCV)

The MCV reflects the average size of an erythrocyte. Its measured unit is expressed in femtoliters (fL) or cubic micrometers (μm^3^). The standard MCV values are 87 ± 7 fL. MCV is used to classify the anemia as normocytic with normal range MCV, microcytic with below the normal range MCV, and macrocytic with above the normal range MCV. The latter also measures RBC distribution width [27,30].

Mean Corpuscular Hemoglobin (MCH)

The MCH reflects the Hb amount per RBC. The normal value of MCH is 29 ± 2 pg per cell [27].

Mean Corpuscular Hemoglobin Concentration

The average Hb concentration per RBC is represented in FBC by the MCHC. Its standard unit is in grams per deciliter of RBCs. A normal MCHC value is 34 ± 2 g/dL [27]. Hyperchromic cells with MCHC >36 are found in hereditary spherocytosis, autoimmune hemolytic anemia, and xerocytosis. Hypochromic cells with MCHC <32 are found in iron deficiency anemia, sideroblastic anemia, and thalassemia [20,31].

Red Blood Cell Distribution Width

RBC size heterogeneity is assessed by RDW and is expressed in percentage. The normal value of RDW is 13% ± 1.5%. The RDW is the ratio of the erythrocyte volume standard deviation to the MCV [27]. A high RDW indicates a wide range of RBC sizes, whereas a low RDW indicates a more uniform RBC population [32].

COPD and anemia

COPD is a complex and heterogeneous lung disease with multifactorial risk factors and variable clinical manifestations [33]. COPD is associated with several distinguishing extrapulmonary comorbidities such as cardiovascular disorders, lung cancer, metabolic disease, reduced bone mass, stroke, cachexia, anemia, and others [34].

An observational study of the valuation of COPD Longitudinally to Identify Predictive Surrogate Endpoints (ECLIPSE) concluded that the prevalence of comorbidities is higher in COPD patients, reaching up to 38%, compared to smokers with normal lung function and non-smokers [35]. In addition, a recent study conducted on COPD patients in a Tunisian Hospital established that comorbidities in COPD patients result in poorer prognosis and higher severity of symptoms [36].

However, recent literature shows that anemia has gained immense significance as a predictor of COPD’s severity, mortality, and prognosis relative to other extrapulmonary comorbidities. Hence, the inter-relationship between anemia and COPD cannot be denied [21,37]. Numerous studies have confirmed that anemia exhibits an independent survival prognostic rate for COPD, negatively impacting the quality of life [21]. Bartolome R Celli et al. studied the various variables responsible for predicting survival in COPD patients. They mentioned that anemia is a major marker of mortality alongside FEV1, lung hyperinflation, and pulmonary cachexia [38].

According to the WHO, anemia is defined as having Hb levels less than 12 g/dL and 13 g/dL in women and men, respectively. However, no specific cutoff value has been assigned to anemia in COPD patients [39].

**Figure 1:**
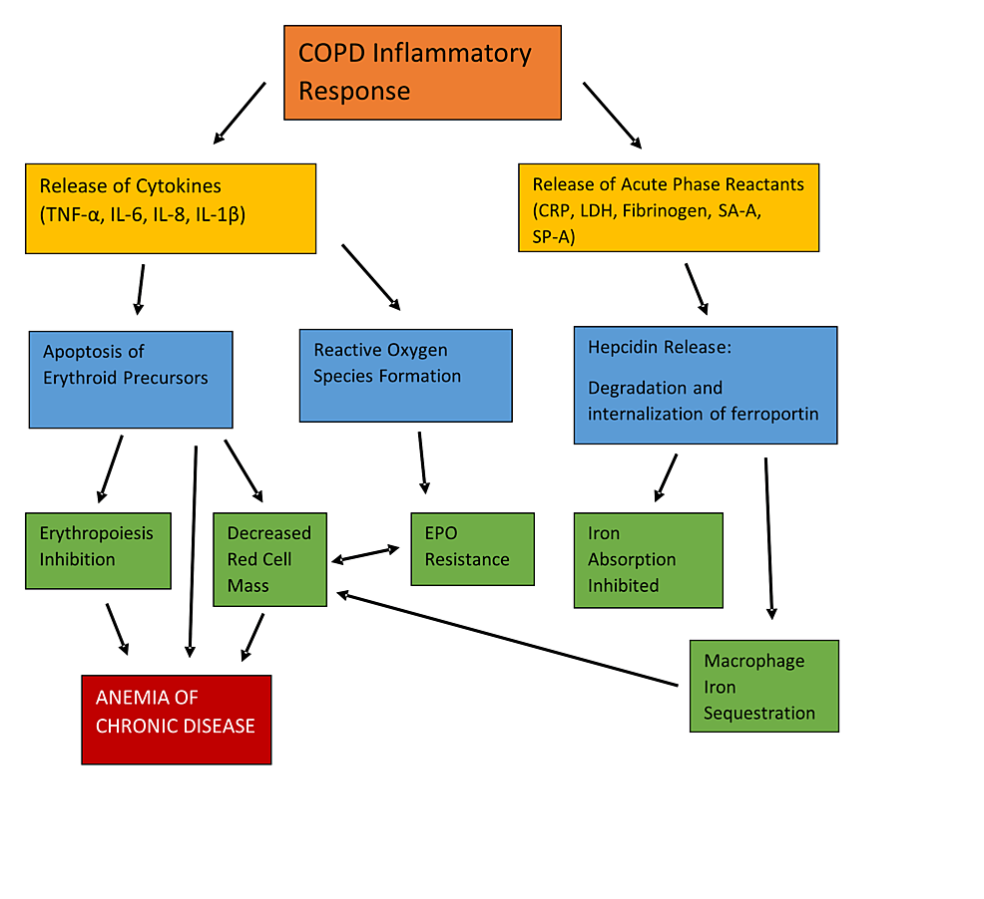
Pathophysiology of anemia of chronic disease in COPD TNF-α: Tumor necrosis factor-alpha; IL-6: Interleukin-6; IL-8: Interleukin-8; IL-1β: Interleukin-1-beta; CRP: C- reactive protein; LDH: Lactate dehydrogenase; SA-A: Serum amyloid-A; SP-D: Surfactant protein-D; EPO: Erythropoietin; COPD: Chronic obstructive pulmonary disease Copyright/License: This figure is recreated using data from an open-access article distributed under the terms and conditions of the Creative Commons Attribution-Non-Commercial 4.0 (CC BY-NC 4.0) license. (http://creativecommons.org/licenses/by-nc/4.0/) Patel MS, McKie E, Steiner MC, Pascoe SJ, Polkey MI: Anaemia and iron dysregulation: untapped therapeutic targets in chronic lung disease?. BMJ Open Respir Res. 2019, 6:e000454. 10.1136/bmjresp-2019-000454 [40].

Different mechanisms lead to the development of anemia in COPD patients. One of them involves the release of acute-phase reactants (CRP, lactate dehydrogenase [LDH], fibrinogen) and the cytokines (tumor necrosis factor-alpha [TNF-α], interleukin-6 [IL-6], interleukin-8 [IL-8], interleukin-1-beta [IL-1β]) due to the inflammatory response in the respiratory pathways ultimately leading to the inhibition of erythropoiesis. However, the blunted erythropoiesis, decreased EPO production, shortened RBC survival, and dysregulation in iron homeostasis eventually result in anemia of chronic disease (Figure 1) [37,39,40].

Anemia of chronic disease is normocytic normochromic anemia occurring in chronic inflammatory diseases such as rheumatoid arthritis, cancer, and chronic kidney disease, most likely due to EPO resistance leading to elevated EPO levels in these patients [40]. 

Other mechanisms involved in developing anemia in COPD patients include renal dysfunction, renin-angiotensin-aldosterone activation by drugs, and hypogonadism [39,41-44].

However, many confounding factors also play a role in the pathophysiology of anemia in COPD patients like cardiovascular disorders, old age, malnutrition, occult blood loss, drugs such as angiotensin-converting enzyme inhibitors or theophylline, endocrine abnormality, and oxygen therapy [36,44-46]. Therefore, screening for other types of anemias, such as iron, folate, or vitamin B12 deficiency, is also necessary for COPD patients [36,45].

Anemic COPD patients have higher rates of hospitalizations and increased healthcare resource utilization than non-anemic COPD patients leading to poor quality of life [21]. A post hoc analysis also showed diminishing health-related quality of life in moderate-to-severe COPD patients with anemia [47]. In addition, a systematic review and meta-analysis in 2020 revealed that anemic COPD patients have a higher mortality rate, Charlson comorbidity index score (predicts ten-year mortality in comorbid patients), and prolonged hospital stays compared to non-anemic COPD patients [33,48].

The comorbidities like anemia also affect and complicate the management of COPD [41,49]. For example, a study conducted by Schonhofer et al. concluded that transfusion of RBCs led to a remarkable reduction in the work of breathing and minute ventilation in anemic COPD patients, and the reduced load on the respiratory muscles improved their dyspnea and exercise capacity. They also found that blood transfusion helped in the successful weaning of ventilated COPD patients with anemia [50].

The use of other treatment options, like EPO therapy and iron supplementation for the treatment of anemia in COPD patients, requires more promising literature reviews [51-53]. The raised EPO levels in anemic COPD patients are a physiologic compensatory mechanism and are possibly related to EPO resistance. Therefore, COPD patients show poor responses to treatment with EPO [54].

Hence, there is an increased need to address the underlying anemia in COPD patients for better clinical outcomes and enhanced survival rates.

COPD and erythrocytosis/polycythemia 

Polycythemia is defined as an Hb level ≥18 g/dL in men and ≥15 g/dL in women [55]. Secondary polycythemia usually occurs due to chronic hypoxia, which increases the production of EPO. EPO is an endogenous glycoprotein hormone that stimulates erythropoiesis. EPO is produced primarily in the kidney, but the liver is another EPO source. EPO stimulates the final differentiation of progenitor cells into erythrocytes in the bone marrow [19,56]. 

The main trigger for EPO formation is a decrease in arterial oxygen content due to anemia or hypoxia, which usually results in an exponential increase in EPO production [6,16]. There is evidence that peritubular cells that secrete EPO contain the heme-containing protein that senses oxygen saturation in the blood [19,57]. As the partial pressure of oxygen (pO_2_) in the plasma decreases, EPO concentration will increase [19,58]. 

Other than hypoxia, polycythemia may be caused by acidosis, whether metabolic (lactic acidosis) or respiratory (chronic respiratory failure) [19,59]. Hypoxia can cause lactic acidosis and produce a vicious circle of inflammation and oxidative stress [19,60].

According to recent studies, polycythemia appears to be less of a problem among today's COPD patients. For example, Cote et al. found a prevalence of only 6% in a prospective cohort of 683 stable COPD outpatients, and only 8.4% of approximately 2,500 patients with severe COPD on long-term oxygen therapy (LTOT) had an HCT of more than 55%. This low prevalence can be partially attributable to the widespread prescription of LTOT in the severe COPD population [38].

While relatively uncommon in the modern COPD population, historical evidence suggests that polycythemia can cause pulmonary hypertension, dysfunction of pulmonary endothelium, decreased cerebral blood flow, hyperuricemia, gout, and a higher risk of venous thromboembolic disease [38]. Polycythemia, which increases blood viscosity, may increase hypoxemia and hypercapnic risks in COPD patients [55]. As with pulmonary hypertension, its presence in a COPD patient should prompt consideration of supplemental oxygen therapy [38].

COPD correlation with RBC indices

COPD and Hb/HCT

Both high and low Hb and HCT indexes are related to COPD, causing different comorbidities.

It is well known that secondary erythrocytosis occurs as a compensatory mechanism in response to hypoxemia seen in COPD patients. However, new research suggests that systemic inflammation in COPD can possibly cause low Hb in these patients [60].

COPD patients with low Hb have a poor quality of life due to reduced exercise tolerance and raised shortness of breath [61].

A study conducted on COPD patients treated with LTOT established that COPD patients with low Hb have a worse prognosis than COPD patients with normal Hb levels. It also indicated that low HCT negatively predicts survival and hospital admission rates [62].

A database study conducted by the French respiratory home care network, the Association Nationale pour le Traitement a Domicile de l'Insuffisance Respiratoire Chronique (ANTADIR), has shown the most promising evidence for the association between HCT and mortality. It states that HCT is inversely related to age and degree of obstruction (FEV1/ vital capacity) but has a positive association with carbon dioxide arterial partial pressure (PaCO_2_). It also emphasizes that polycythemia had higher survival rates (three-year survival 24%) when HCT was <35% compared to when HCT was <55% (three-year survival 70%) [62].

Treatment of low and high Hb concentrations has a significant clinical impact on the prognosis of COPD patients. A rise in hemoglobinemia through a blood transfusion improves skeletal muscle function, breathing pattern as well as pulmonary gas exchange, which alleviates dyspnea and enhances exercise capacity [63].

Another study demonstrated that oxygen administration to severe COPD patients before exercise improves exercise tolerance rates [64]. This phenomenon is similar to raising Hb levels by infusing RBC transfusion in COPD patients to decrease the degree of hyperinflation and improve symptomatology [63].

In COPD patients, the incidence of increased HCT and Hb levels (polycythemia) is immensely reduced due to the implementation of close follow-up and LTOT, whereas low Hb (anemia) has become a concern nowadays [65]. Furthermore, two other studies highlighted that in patients with an HCT level of 50%-55%, phlebotomy had improved the hemodynamic response to exertion in COPD patients due to reduced pulmonary arterial resistance and arteriovenous oxygen content difference [66].

Therefore, it is determined that low Hb has more of a close association with survival and mortality outcomes of COPD patients than high Hb levels. A piece of well-established evidence is available on the correction of raised Hb levels in COPD patients, whereas the treatment for low Hb levels (anemia) requires further data exploration.

*COPD and MCHC* 

MCHC indicates the Hb concentration within each RBC. As a result, low MCHC specifies functional iron shortage. Reduction in functional iron levels can be caused by systemic inflammation, such as in COPD, an inflammatory lung disease [8]. Anemia is caused by iron deficiency; however, non-anemic iron deficiency can occur in patients who have not been tested for anemia. In this case, MCHC is also low; thus, iron deficiency may occur before the expression of anemia in COPD [8]. 

The exact mechanisms behind the link between MCHC and chronic illness prognoses, such as COPD or heart disease, are unknown. According to prior studies, MCHC represents iron deficiency, and chronic inflammation is one of the reasons for iron deficiency. Therefore, the decrease in MCHC may reflect the intensity of inflammation [7]. In a 2021 study by Kanwal et al., the MCHC and COPD were significantly associated (p=0.03) [22].

COPD and RDW

The RDW is typically reported in the complete blood count (CBC) as a marker of erythrocyte size heterogeneity. However, its most considerable role resides in the differential diagnosis of anemia, along with the MCV and MCH [67]. An increased RDW is an indicator of anisocytosis, mainly seen in iron deficiency, vitamin B12, or folate deficiency anemia. However, chronic disease anemia, aplastic anemia, congenital spherocytosis, acute blood loss, and some hemoglobinopathies are all linked to a normal RDW [68].

Recently, RDW elevation has been linked to several disorders, including cardiovascular illness, cerebrovascular disease, pulmonary embolism, cancer, diabetes, acute kidney failure, and others. Furthermore, RDW is thought to be a strong and independent risk factor in predicting mortality [69]. According to these studies, higher RDW levels could indicate an underlying chronic inflammation, which promotes erythropoiesis disturbances and RBC membrane deformability [9]. Abnormal erythrocyte survival, telomere shortening, oxidative stress, hypoproteinemia, dyslipidemia, hypertension, erythrocyte fragmentation, and EPO function alterations are other factors to consider [68]. 

COPD also causes systemic inflammation, which has been suggested as a crucial factor in the link between COPD and elevated RDW. This shared feature has been the trigger behind the theory inspiring multiple research studies on RDW as a negative prognosis factor of COPD [69].

According to a recent study, patients with COPD exhibited considerably greater RDW values than control participants (15%±2.3% vs. 13.8%±2.5%, p<0.001). In COPD patients, RDW levels also correlated positively with CRP levels (r=0.27, p<0.01), albumin levels (r=0.23, p=0.04), right ventricular dysfunction (RVD) (r=0.24, p=0.01), pulmonary arterial hypertension (r=0.1, p=0.02), and cardiovascular disease (CVD) (r=0.24, p=0.02). Otherwise, RDW levels were inversely correlated with Hb concentration (r=−0.38, p=0.01). More importantly, RDW was independently associated with CVD and RVD in patients with COPD [9].

In another study, the severity of COPD was relatively proportionate to an increase in mean RDW levels (Table 1) [9].

**Table 1:** RDW levels correlation with GOLD COPD stages (p<0.001) RDW: Red cell distribution width; GOLD: Global initiative for chronic obstructive lung disease. Copyright/License: This figure is from an open-access article distributed under the terms and conditions of the Creative Commons Attribution-Non-Commercial-NoDerivatives 4.0 (CC BY-NC-ND 4.0) license. (https://creativecommons.org/licenses/by-nc-nd/4.0/) No modifications were made to the original figure. Tertemiz KC, Ozgen Alpaydin A, Sevinc C, Ellidokuz H, Acara AC, Cimrin A: Could "red cell distribution width" predict COPD severity?. Rev Port Pneumol (2006). 2016, 22:196-201. 10.1016/j.rppnen.2015.11.006 [9].

GOLD Stages	Mean RDW (%)
Stage 1	13.5
Stage 2	13.9
Stage 3	14.4
Stage 4	15.7

**Figure 2:**
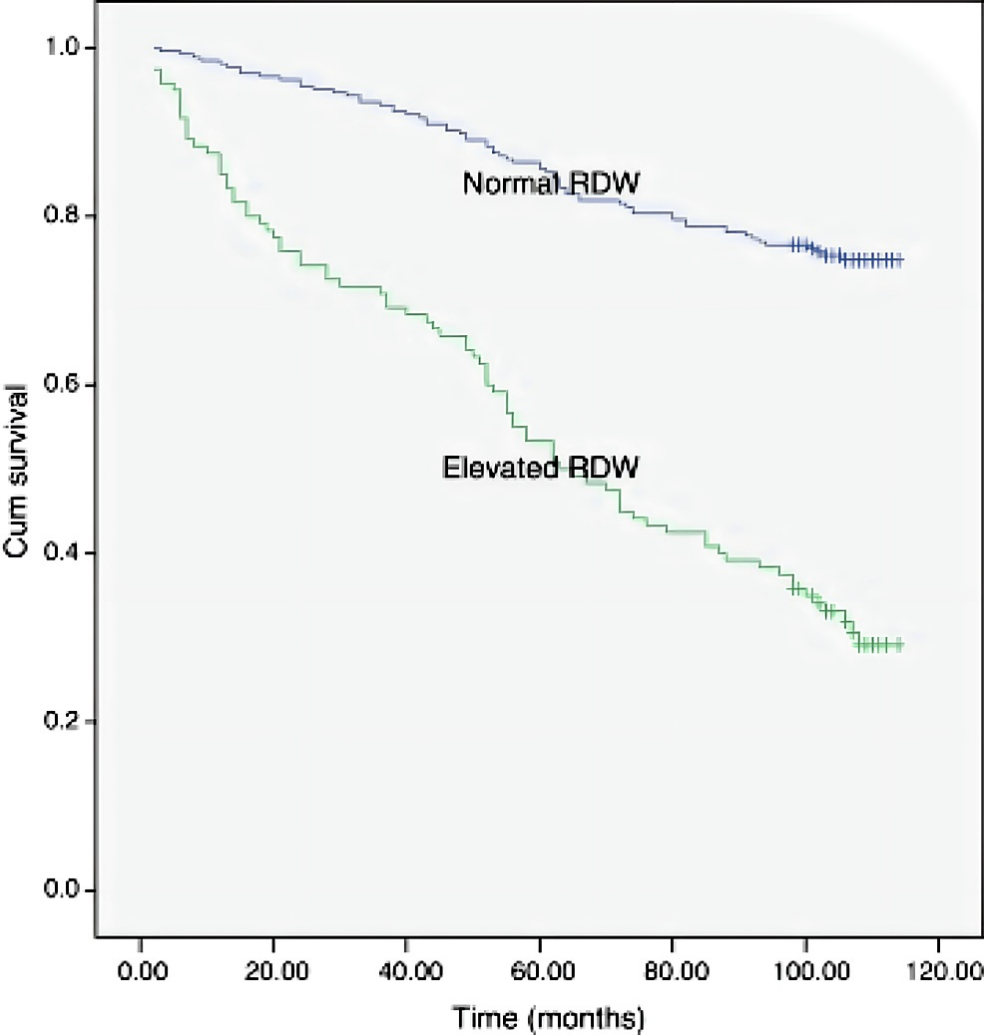
Representation of nine-year survival of COPD patients according to RDW in a Kaplan-Meier curve (p<0.01) RDW: Red cell distribution width; COPD: Chronic obstructive pulmonary disease. Copyright/License: This figure is from an open-access article distributed under the terms and conditions of the Creative Commons Attribution-Non-Commercial-NoDerivatives 4.0 (CC BY-NC-ND 4.0) license. (https://creativecommons.org/licenses/by-nc-nd/4.0/) No modifications were made to the original figure. Tertemiz KC, Ozgen Alpaydin A, Sevinc C, Ellidokuz H, Acara AC, Cimrin A: Could "red cell distribution width" predict COPD severity?. Rev Port Pneumol (2006). 2016, 22:196-201. 10.1016/j.rppnen.2015.11.006 [9].

Patients with an increased RDW also had decreased pulmonary functional parameters, a six-minute walking test (6MWT) distance, and oxygen saturation. High RDW levels in the same patients were associated with increased age, smoking, and BODE index (Body mass index, Obstruction, Dyspnea, Exercise capacity), which is another COPD prognosis factor [70]. Additionally, COPD patients with a normal RDW (14.3%) had a 75% nine-year survival rate, while patients with a high RDW (>14.3%) had a 31% survival rate (Figure 2) [9].

RDW was also included in the studies evaluating acute exacerbations of COPD (AECOPD) severity and mortality with the requirement of several therapies in the treatment of respiratory failure. Patients who had been hospitalized in the previous 12 months showed higher RDW values than those who had not (p<0.01) [70]. Patients in need of non-invasive mechanical ventilation (NIMV) had a substantially higher median RDW than patients who did not need NIMV (p<0.001). Patients who needed LTOT also had a significantly higher median RDW (14.2, 95% CI: 13.7-14.6) than patients who did not need LTOT (p=0.001) [71]. 

In another study, the 30-day all-cause readmission of patients with AECOPD was independently linked with dynamic increases in RDW (p=0.008) [72].

Concerning the mortality of patients with AECOPD, RDW has a major prognosis role as it was demonstrated that RDW ≥13.75% was a risk factor for in-hospital mortality and independently correlated with death at one year after an AECOPD [73]. 

COPD and RBC structural alterations 

The pathophysiologic mechanisms of COPD are very intricate; however, localized pulmonary and systemic inflammatory responses with associated oxidative stress were not only significant contributors to the disease but were associated with its progression and studied as markers of advanced stages [74,75]. These phenomena would cause a fundamental imbalance between pro-oxidants and antioxidants with increased generation of ROS and reactive nitrogen species capable of damaging DNA, lipids, carbohydrates, and proteins [76]. Of course, as an essential compound of our system, RBCs will not be exempt. As a result, chronic oxidative stress will directly damage erythrocytes resulting in structural and functional alterations [77]. 

Erythrocytes serve as oxygen transporters and deliverers. They also have powerful antioxidant systems that enable them to act as mobile free radical scavengers, protecting not just themselves but also other tissues and organs in the body [77]. Thus, it stands to reason that if their primary structure and enzymes are compromised, oxygen exchange and transport will be altered, contributing further to hypoxemia induced by the destruction of the blood-gas barrier in COPD [11]. Oxidative stress and damage will be accentuated as RBCs’ antioxidant properties are significantly reduced, leading to a vicious cycle of RBC injuries and severe COPD disease progression [76].

In COPD patients, multiple studies have demonstrated the specific effects of inflammatory and oxidative reactions. Bożena Bukowska et al. showed evidence of an increase in lipid peroxidation products with a decrease in the quantity of sulfhydryl or thiol groups in the erythrocytes membrane. Moreover, glutathione peroxidase activity was increased in contrast to superoxide dismutase activity. Other significant alterations were also observed, as evidenced by a substantially reduced adenosine triphosphatase activity and increased acetylcholinesterase activity, key enzymes for erythrocyte structure and function [78].

Another study conducted on patients with moderate-to-severe COPD demonstrated a decreased oxidation of glucose-6-phosphate dehydrogenase, glutathione reductase, and glutathione peroxidase [75]. It supports substantial damage to RBCs with decreased function; however, erythrocyte integrity was still preserved, enabling patients to live without hemolysis [75].

Studies on erythrocyte structural changes illustrated increased RBC spheronization with augmented platelet migration to the vessel wall. This could explain why COPD patients have such a high rate of cardiovascular events [79]. 

During COPD exacerbations, RBC deformability was proven to be decreased with associated increased aggregation capacity, which may worsen patients’ oxygenation and clinical symptoms [80].

## Conclusions

Although multiple factors have been assessed in understanding COPD mortality/morbidity and treatment monitoring strategies, COPD and RBC indices correlation is still undeniable. We concluded that chronic systemic inflammation, chronic oxidative stress, and impaired iron metabolism are the main pathologies associated with COPD that alter RBC indices. Ongoing systemic inflammation affects the structure and function of erythrocytes, reducing their deformability and interference with erythropoiesis. Low Hb, HCT, and MCHC, and high RDW levels were associated with poor prognosis, lowering survival, and raising mortality. RBC indices were also studied for use in guiding COPD treatment. Patients with a higher RDW had more hospitalizations and required LTOT therapy. Therefore, we recommend considering RBC indices as a prognostic indicator in assessing disease severity, treatment, and follow-up of COPD patients to reduce exacerbation episodes and hospital readmissions.
